# Why do we continue to have an incomplete understanding of estrogen receptor(s) actions in cancer systems?

**DOI:** 10.1530/ERC-25-0036

**Published:** 2025-05-05

**Authors:** Hamish D McMillan, Jason S Carroll

**Affiliations:** CRUK Cambridge Institute, University of Cambridge, Cambridge, UK

**Keywords:** estrogen receptor, genomics, cancer

## Abstract

The topic proposed asks why we have an incomplete understanding of estrogen receptor (ER) action in cancer. We agree that the role of ER in some cancers is poorly understood, but the role of ER in luminal breast cancer (which represents the biggest subtype of the most common cancer in women) is very well characterised. Although technologies evolve and allow us to learn more about the intricacies of the genome and the multitude of ways breast cancer can adapt to bypass treatments, our understanding of how ER regulates gene expression is extraordinary. We would posit that the key events in ER-mediated cell cycle progression are already known and although new information adds granular information to our paradigms of ER action, we need to better characterise what has been discovered already.

## Background

There are specific features about estrogen receptor (ER) positive breast cancer that make it amenable for biological characterisation but also make it a consistent clinical problem. ER-alpha is the driving transcription factor in 80% of all breast cancers (Giaquinto *et al.* 2024). The second ER protein, ER-beta, is either not expressed in breast cancer or expressed at levels too low for detection by traditional methods (Monteiro *et al.* 2024) and as such, ER-alpha (from herein ER) is the more abundant and clinically validated ER in this disease. More than three out of every four breast cancer cases are defined by and driven by the ER transcriptional pathway, which in total constitutes approximately 248,000 new cases in the US each year (Giaquinto *et al.* 2024). What is unusual about this breast cancer subtype is that ER+ disease is mediated by a single transcriptional pathway and dysregulation of this pathway can occur independently of mutational events. Given the critical role for ER in mammary gland development, it is possible that ER+ disease arises from reinitiation of a pre-existing but latent transcriptional pathway at the wrong time in life, although we know that the mechanisms of action are not the same, suggesting cancer-specific events are responsible for restarting ER transcriptional activity later in life.

A myriad of treatments have been developed for ER+ breast cancer patients and these have extended survival substantially, with 5-year survival rates now greater than 90% for localised disease (Giaquinto *et al.* 2024). Despite the success of treating ER+ cancer, two key issues exist. The first is that almost all existing treatments target ER directly or indirectly through a variety of mechanisms. These include direct modulators of ER such as selective ER modulators (SERMs) and selective ER degraders (SERDs), which directly bind ER to modulate its transcriptional capacity and/or degrade the protein. Indirect modulators of ER activity, such as aromatase inhibitors, work by inhibiting the metabolism of estrogen from the precursor molecule, thereby decreasing the activating ligand for ER to prevent ER-driven transcription. Alternatively, targeting of critical downstream ER target genes with CDK4/6 inhibitors, which prevent ER-driven cell cycling, provides an ER-independent mechanism of targeting ER-driven growth. The targeting of the same pathway, even at multiple points in the molecular process, means that treatment escape mechanisms might bypass multiple treatment modalities simultaneously. The second key issue (which might be a consequence of the first issue) is that despite improvements in overall survival, ER+ breast cancer patients continue to relapse, often with metastatic disease, sometimes decades after diagnosis. Our understanding of the biology of ER+ disease has evolved at an extraordinary rate, but there are still major unresolved issues that need to be addressed, and these will be discussed in this review.

Despite the title suggesting that we, as a community, have an incomplete understanding of how ER works in cancer, we would argue that we have an extraordinary understanding of how ER can work in primary breast cancer but we lack understanding in cancer types such as ovarian cancer and in the advanced disease setting, where it matters the most. We would argue that we know more about ER action in primary ER+ breast cancer contexts than almost any other transcriptional process and that the study of ER has been critical to our understanding of gene regulation and transcription as a whole. Traditionally, breast cancer has received substantial attention because of the prevalence of the disease and the proactive fundraising that underpinned the research. Despite the fact that robust ER+ genetic models are lacking in mice (although there have been substantial unsuccessful attempts at creating a reproducible genetic model of ER+ disease) and despite the fact that very few cell line models exist that accurately represent ER+ primary disease, the field has benefited from one major variable. This variable is the hyper-dependence on estrogen for activation of ER, which means that cell line models can be synchronised. As ER is the central driver of cell cycle-promoting transcriptional pathways and by extension cell proliferation, its inactivation by the removal of ligand through hormone deprivation allows the synchronisation and arrest of cells in the G1 phase of the cell cycle (Renoir *et al.* 2008). Estrogen deprivation dramatically reduces the levels of transcription within the cell, resulting in cell cycle arrest in G1/G0. The subsequent treatment of deprived cells with estrogen results in a synchronised wave of transcriptional activity where molecular events are synchronised throughout the cell population and therefore amplified. This ‘on-off’ ligand-inducible switch provides an attractive model for characterising the fundamental properties of transcription factor biology that result from ER activation. Much of what we know about basic principles of genomic regulation during transcription was first gleaned from these estrogen-stimulated experiments before being further characterised and tested in other models. The vast majority of these insights have been derived from a single cell line model, namely the MCF7 cell line.

By exploiting the ligand-inducible system in the ER+ MCF7 breast cancer model, the community has learnt an extraordinary amount about the complexities of ER activity and transcriptional regulation in general. Our understanding of how ER docks onto the DNA and the relevant motifs that it has affinity for were derived, in part, from studies of this cell line. The target genes of the ER transcriptional pathway were identified by characterising the genes that were induced following short-term estrogen stimulation of estrogen-deprived cells. The direct ER target genes that were identified from these simple *in vitro* models were subsequently validated as the key signature genes of ER+ breast cancer (Perou *et al.* 2000). Some of these target genes have been incorporated into commercial gene expression diagnosis platforms used for predictive treatment decisions, since they represent a robust signature of the ER+ (luminal) breast cancer subtype. Importantly, it has been known for more than two decades that two key ER target genes are sufficient to promote cell cycle progression. These two genes are c-Myc and cyclin D1, both of which are direct ER target genes and both of which can phenocopy ER activity (Wilcken *et al.* 1997, Prall *et al.* 1998). These findings suggest that the ability of ER to promote cell cycle progression and therefore cancer progression results almost entirely from the fact that ER directly turns on cyclin D1 and c-Myc. The fact that the essential kinase partner of cyclin D1 is CDK4 likely explains the specific sensitivity of ER+ breast cancer patients to CDK4/6 inhibitors. Since cyclin D1-CDK4/6 signalling as a key downstream event of ER transcription likely explains the effectiveness of these CDK4/6 inhibitors both in combination with hormone therapy, where they provide a second mechanism to block ER-driven proliferation, and in metastatic breast cancer after hormonal and chemotherapy have failed.

## Technological advances in genomics have revolutionised our understanding of ER biology

The advent of tiling microarrays and subsequently massively parallel sequencing was transformative to biological science and, in particular, in characterising the genomic variables that can influence and are influenced by ER transcriptional activity. The existence of high-quality ER-specific antibodies meant that chromatin immunoprecipitation (ChIP) experiments could readily be conducted to map ER-chromatin interactions. The development of tiling microarrays that covered entire chromosomes and ultimately the entire non-repetitive genome resulted in one of the first genome-wide maps of any transcription factor (Carroll *et al.* 2005, 2006, Tollkuhn 2024). Estrogen-stimulated MCF7 cells were used to map ER binding sites in an unbiased manner, resulting in the observation that ER does not bind to promoter-proximal regions and instead associates with distal enhancers and it also revealed the role of associated transcription factors such as GATA3 and FOXA1 (Carroll *et al.* 2006). These findings revealed the concept of pioneer factors in cancer, where proteins such as FOXA1 work with nuclear receptors such as ER to create *de novo* enhancer elements. The subsequent identification of specific histone modifications at these regulatory elements (Lupien *et al.* 2008, Sérandour *et al.* 2011) helped define the epigenetic properties associated with enhancer elements, the variables that contribute to enhancer elements (Jozwik *et al.* 2016) and the clinical impact of genetic variants in these regions (Cowper-Sal lari *et al.* 2012). A major unresolved issue is how ER binding changes from healthy, non-cancerous tissue to cancer. The mapping of ER from healthy mammary gland tissue (i.e., reduction mammoplasties) is required to do this comparison and although attempts have been made, none of the data is publicly available yet.

The application of these genome-wide mapping approaches to clinical material has further improved our understanding of how ER drives primary breast cancer growth and provides insight into its role in determining patient outcome. The identification of ER binding, sites which predict good and poor patient outcome and the observation that these are conserved in metastasis provided further insight into the function of ER in breast cancer (Ross-Innes *et al.* 2012). Despite the core function of ER as a driver of ER+ breast cancer proliferation, there is substantial heterogeneity in ER binding between patients. As little as 30% of ER binding sites are conserved between patients, however these binding sites are enriched for functionality, with target gene activation substantially more conserved across patients, even if the adjacent regulatory elements are different between patient samples. These results suggest a convergence across patients of ER functionality in promoting a conserved transcriptional programme but suggest that ER may be able to achieve this programme through the use of a variety of different binding sites (Joosten *et al.* 2024).

As genomic techniques evolved, these were consistently utilised for the first time in MCF7 cells using the minus/plus estrogen switch. As two major examples, the concept of transcription factor-mediated chromatin loops and the identification of global chromatin interactions between regulatory elements was established with the initial ChIA-PET studies (Fullwood *et al.* 2009), which revealed extraordinary complexity in the different chromatin loops that could be formed between ER regulatory elements in a breast cancer genome. This ChIA-PET study of ER and its continued analysis have revealed the convergence of this complex collection of adjacent regulatory chromatin interactions, often involving a range of enhancers at the core ER-regulated genes. The second major genomic concept that was derived from this cellular system was the discovery of enhancer RNAs and the substantial amount of non-coding RNA that could be detected using nascent transcriptomic mapping (Hah *et al.* 2011, Franco *et al.* 2018, Hou & Kraus 2022). These findings suggested that the ER transcription complex could induce transcripts from a significant portion of the non-coding genome, redefining the number of transcriptional targets induced by the ER complex. The nascent transcripts, including enhancer RNAs (eRNAs), produced from the ER transcriptional complex have been associated with a range of functions, including transcriptional regulation through the promotion of chromatin loops and co-factor recruitment, although the role for eRNAs in mediating chromatin loops has been questioned, with others not finding a functional link between eRNAs and chromatin loops (Hah *et al.* 2013). These findings have helped define fundamental properties of transcriptional regulation and have revealed an unexpected degree of plasticity in the genome with regard to how regulatory elements can be used and what can be transcribed.

## Limitations of current genomic technologies

A major limitation in the field is the dependence on a small number of cellular models that are typically grown *in vitro*. Although the genomic datasets from these models have been incredibly informative and have provided useful databases for the community to mine (Li *et al.* 2023, 2024), the field has been limited by the fact that we are still studying the ‘average of averages’. ChIP-seq style experiments provide insight into the average binding intensity at specific loci in the genome. Comparison of multiple independent ChIP-seq experiments would suggest that key ER-associated proteins typically bind to the same regions in the genome, near the target genes of interest. What is not clear is whether these proteins bind to the same sites, at the same time, in the same sub-population of the cells. One possibility is that co-binding, as defined by co-localised ChIP-seq signal, could in fact represent mutually exclusive binding patterns that come from distinct subsets of the cell population: i.e., that two factors only bind to the same locus at different times and therefore in different subsets of the cell population. These bulk ChIP-seq approaches would therefore inaccurately suggest that these two proteins are binding together and could be functionally connected, whereas in reality, they might never associate together and there might in fact be a negative regulatory connection. Until single-cell transcription factor mapping approaches are established, we are unable to decipher this level of complexity but, with rapidly evolving single-cell technologies, we hope that single-cell ChIP-seq (or equivalent methods) will be available in the coming years. This information could be integrated with single-cell RNA-seq data to identify the distinct ER binding events that exist within a population of cells and the associated transcriptional events.

## Mass spectrometry based approaches have revealed the dynamic co-factor networks associated with ER

Numerous ER-associated co-factors have been identified over the decades, many of which are well-established chromatin-regulatory enzymes that can inhibit or promote ER transcriptional activity. Unbiased IP-mass spectrometry based approaches, such as RIME (Mohammed *et al.* 2013, 2016, Papachristou *et al.* 2018) have revealed many more ER-associated proteins. Although all of the validated ER-interacting proteins can associate with ER, it is not clear what the different protein subcomplexes are. It is unlikely that all associated co-factors interact with ER at the same time and a more likely scenario is that distinct, functionally unrelated subcomplexes exist with ER. Given the rapid cycling of ER onto and off the chromatin (Shang *et al.* 2000, Métivier *et al.* 2003), a phenomenon that is also seen with glucocorticoid receptor (Nagaich *et al.* 2004, Stavreva *et al.* 2004, Paakinaho *et al.* 2017), it is likely that different protein subcomplexes exist for rapid ER recruitment and clearance from the chromatin. The specific complexes formed by ER are important to function and even subtle changes in the complex can have significant impacts on the transcription driven by ER. For example, TET2 is a core member of the ER complex but its loss leads to minimal disruption in the overall ER transcriptional complex. However, TET2 loss decreases activation of proliferative transcriptional pathways in breast cancer cells by altering the 5-hydroxymethylcytosine levels specifically at ER bound sites (Wang *et al.* 2018, Broome *et al.* 2021). However, as TET2 is not observed at all ER bound sites, this suggests that this mechanism of transcriptional regulation is specific to certain genomic locations and a specific ER subcomplex.

An alternative regulator of ER transcription is ARID1A, which has been shown to associate with ER and aids in the maintenance of the luminal phenotype of ER+ breast cancer cells (Nagarajan *et al.* 2020, Xu *et al.* 2020). The loss of ARID1A leads to endocrine therapy resistance in both cell line models and patients, further suggesting a strong link between ARID1A and ER in driving transcription. Interestingly, despite the co-localisation of ER and ARID1A and the known association, the recruitment of ARID1A is thought to be driven by ER’s pioneer factor FOXA1 (Nagarajan *et al.* 2020). Finally, interaction between ER and different nuclear receptors is also critical in determining ER localisation and the transcriptional programmes it can drive. Both androgen and progesterone receptors have been shown to interact with ER and alter both ER complex formation and chromatin binding (Mohammed *et al.* 2015, Hickey *et al.* 2021). Given the nature of hormone signalling as concurrent processes due to the presence of multiple hormone receptors in the same cellular contexts, our understanding of the interplay between ER and other nuclear receptors is important for understanding disease and where we could exploit existing nuclear receptor agonists and antagonist ligands.

The aforementioned examples highlight a key issue in the field which is identifying which protein subcomplexes exist and how they are functionally related to one another. Identifying critical subcomplexes with specific functions could open a number of treatment options as the number of potentially clinical-grade inhibitors against ER-associated proteins is increasing at a rapid pace. Inhibitors against known functional ER-associated proteins exist, including inhibitors against p300/CBP (Zou *et al.* 2019, Nicosia *et al.* 2023), LSD1 (Fang *et al.* 2019, Nicosia *et al.* 2022), bromodomain proteins (Wang *et al.* 2023), PRMT family member (Hwang *et al.* 2021), histone deacetylase proteins (Bondarev *et al.* 2021) and TET2 (Kaplánek *et al.* 2023). Many of these proteins are generic chromatin regulatory factors, meaning that treatment-associated toxicities are inevitable as these enzymes work with a range of transcription factors across a range of cell types. The systemic delivery of drugs targeting key enzymes that work with ER may also result in inhibition of that enzyme in the multitude of cell types that express and require that protein. These limitations may be partially overcome through targeted delivery approaches, such as antibody drug conjugates. However, a more logical and feasible approach may be the combination of inhibitors that work in a synergistic manner, such that low-dose combinations of inhibitors could have efficacy without the systemic toxicities associated with higher doses of either inhibitor alone. Rational combinations require a deeper understanding of how the ER-associated proteins interact, how these interactions are unique to pathogenic ER, what redundancy mechanisms exist and what co-dependencies exist between co-factors. Key to this is the need for characterisation of ER-associated protein subcomplexes, which may be aided by the deployment of mathematical systems biology type approaches aimed at modelling protein complexes and the effect of drug treatment (He *et al.* 2023). Given the high efficacy of ER-targeted therapy, understanding how protein subcomplexes change under these treatments to allow resistance and what vulnerabilities are created is of particular importance.

## The role of ER in late disease and metastasis requires further investigation

Our ability to characterise ER transcriptional activity in primary tumour models has been extraordinary. We know that ER can associate with hundreds of transcription factors and co-factors and that these complexes can then bind to thousands of regulatory elements within the genome to ultimately regulate many thousands of different transcriptional targets. What we know less about is what changes occur in treatment-resistant disease. Until only a decade ago, ER was thought to be wild type, even in treatment-resistant contexts. While ER mutations were identified in patient samples as early as the late 1990s (Zhang *et al.* 1997), the clinical relevance of such mutations was thought to be minimal as they were thought to be extremely rare. Individual ER mutations had been observed and characterised in specific cell line models but nobody appreciated the frequency of ER (ESR1) mutations in treatment-resistant disease because large studies such as TCGA breast and METABRIC were not looking at the right samples. In 2013, simultaneous observations were made revealing frequent mutations in ER, specifically in metastatic samples resistant to hormonal therapy (Merenbakh-Lamin *et al.* 2013, Robinson *et al.* 2013, Toy *et al.* 2013). Across these three original studies, the prevalence of ER activating mutations was between 17.5 and 54% in metastatic samples, with the same mutations rarely identified in primary tumour samples, matched or otherwise (Merenbakh-Lamin *et al.* 2013, Robinson *et al.* 2013, Toy *et al.* 2013). Even with the relatively low sample numbers of these original studies, a clear trend was evident, ER was seen to be consistently activated by mutations in the ligand binding domain: a selection process that occurred in response to aromatase inhibitor treatment, highlighting the importance of using the most relevant samples.

The different ESR1 mutations and the functional explanation for why these specific mutations provide a growth advantage in the presence of an ER-targeted drug have been described in detail in other articles (Jeselsohn *et al.* 2015, Herzog & Fuqua 2022, Will *et al.* 2023). What these findings clearly demonstrated was that the reason we struggle to manage treatment-resistant disease was because we had not adequately characterised samples from treatment-resistant patients. Large genomic sequencing projects have subsequently been undertaken and we are now aware of the mutations, copy number changes and transcriptional alterations that can contribute to advanced ER+ disease (Lefebvre *et al.* 2016, Li *et al.* 2021, Garcia-Recio *et al.* 2023). The existence of a variety of treatment-resistant gene fusion events (Veeraraghavan *et al.* 2016) has also been identified and a goal for the field is to characterise the functional relevance of these events and to develop approaches to treat patients with these specific genomic or transcriptomic alterations.

Although we have a better understanding of events that are enriched in metastases, we have struggled to model these. As an example, the observation of ESR1 mutations motivated a number of labs to create cell line models with the ESR1 hotspot mutations. Different technical approaches were taken to create models with specific ESR1 mutations and the conclusions from the various investigations were surprisingly different. Some publications concluded that ESR1 mutations created new ER genomic binding sites (Martin *et al.* 2017), whereas others suggested that the mutant ER binding sites were the same as wild type ER binding but simply could occur in ligand-independent conditions (Harrod *et al.* 2022). Conclusions need to be derived from combinations of different models and the community has been proactive about sharing resources and insight to reach clinically meaningful conclusions and new treatment strategies have subsequently been devised for patients with mutant ESR1 cancers.

One of the biggest obstacles in the field was the lack of robust, physiologically-relevant *in vivo* models to study the metastatic process. Typically, ER+ breast cancer cell lines (or single cell PDX suspensions) could be implanted in the mammary fat pad, subcutaneously or via tail vein injection (Zhang *et al.* 1999, Holen *et al.* 2016, Özdemir *et al.* 2018, Jin *et al.* 2020). These models could metastasise to the lung but this did not reflect the metastatic disease seen in patients where metastases are typically observed in the bone, liver, lung and brain. Pioneering work from the Medina lab (Behbod *et al.* 2009), which was refined by the Brisken lab (Sflomos *et al.* 2016), showed that intraductal injection of ER+ breast cancer cells (i.e., into the mammary ducts of mice rather than the fat pad) could accurately recapitulate the metastatic process observed in women, with slow-growing tumours at endogenous estrogen levels, that would metastasise to the same four secondary organs seen in patients with ER+ breast cancer. This intraductal (or MIND) approach has revolutionised our ability to model the metastatic process in ER+ disease. Ongoing work using this model to characterise clinically observed mutations or as a basis for unbiased CRISPR screening approaches will change our understanding of how metastasis evolves and it will reveal new insight into how to more effectively combat treatment-resistant ER+ breast cancer. The MIND model has also opened a door to better understand the dormancy phenomenon observed in ER+ breast cancer, where patients can relapse, often with metastatic disease, 10 or more years after primary disease resection and endocrine therapy. How these cells persist at distant organs for years, often under therapy, and then begin proliferating to form metastatic tumours and the role of ER in this process is a pertinent clinically-relevant question for the field to answer.

## Conclusions

Our understanding of how the transcription factor ER can interact with the genome to turn genes on has changed enormously in the past two decades. The complexities associated with describing each stage in the transcriptional process have been surprising and daunting. That said, we have a solid grasp on what the key ER-associated proteins are, what the critical target genes are and what the important regulatory mechanisms are that ER uses. We still need clarity on some of these mechanistic events and this will come with time. What we also need to understand is what happens when disease progresses and becomes treatment-resistant. The development of better *in vivo* models to study metastasis and improved insight into the clinically observed events that occur in metastasis will almost certainly reveal important information and opportunities. What the field is not lacking is treatments. There are many different ER-targeted drugs and emerging treatments that target peripheral or interconnected pathways in ER+ breast cancers (such as PIK3, mTOR and AKT). What we need to understand is what the best combination of treatments is for the right patients at the right time of the disease. Improved biological insight will inevitably make this possible with time, unmasking even greater levels of complexity hidden within every ER+ cancer cell genome.

To go back to the original topic, we would argue that we likely do have an incomplete understanding of ER activity in breast cancer. That said, what we do know is extraordinary and reflects the exceptional complexity of how a developmental transcription factor like ER can utilise genomic regulatory elements and associated variables to control gene expression. The more we look, the more we will identify and the deeper our understanding will become. We hypothesise that we know the key events in ER-mediated gene regulation, but what we need to better understand is how to use the existing data in a more meaningful manner to predict patients’ responses and to understand and exploit insight into treatment-escape mechanisms.

## Declaration of interest

The authors declare that there is no conflict of interest that could be perceived as prejudicing the impartiality of this review.

## Funding

Jason Carroll is funded by Cancer Research UK Core funds (Grant number: G101107), Hamish McMillan is funded by Breast Cancer Foundation NZ (Grant number: F2201) and Wellcome Trust Discovery Award (Grant number: G122658).
